# Thermal Properties and Crystallite Morphology of Nylon 66 Modified with a Novel Biphenyl Aromatic Liquid Crystalline Epoxy Resin

**DOI:** 10.3390/ijms141020682

**Published:** 2013-10-15

**Authors:** Zhiqi Cai, Shuang Mei, Yuan Lu, Yuanqi He, Pihui Pi, Jiang Cheng, Yu Qian, Xiufang Wen

**Affiliations:** School of Chemistry and Chemical Engineering, South China University of Technology, Guangzhou 510640, China; E-Mails: cezqcai@scut.edu.cn (Z.C.); jusla@163.com (S.M.); l.yuan22@mail.scut.edu.cn (Y.L.); yq.he@100steps.net (Y.H.); phpi@scut.edu.cn (P.P.); cejcheng@scut.edu.cn (J.C.); ceyuqian@scut.edu.cn (Y.Q.)

**Keywords:** liquid crystalline epoxy resin (LCER), Nylon66, Thermal properties, POM

## Abstract

In order to improve the thermal properties of important engineering plastics, a novel kind of liquid crystalline epoxy resin (LCER), 3,3′,5,5′-Tetramethylbiphenyl-4,4′-diyl bis(4-(oxiran-2-ylmethoxy)benzoate) (**M1**) was introduced to blend with nylon 66 (**M2)** at high temperature. The effects of **M1** on chemical modification and crystallite morphology of **M2** were investigated by rheometry, thermo gravimetric analysis (TGA), dynamic differential scanning calorimetry (DSC) and polarized optical microscopy (POM). TGA results showed that the initial decomposition temperature of **M2** increased by about 8 °C by adding 7% wt **M1**, indicating the improvement of thermal stability. DSC results illustrated that the melting point of composites decreased by 12 °C compared to **M2** as the content of **M1** increased, showing the improvement of processing property. POM measurements confirmed that dimension of nylon-66 spherulites and crystallization region decreased because of the addition of liquid crystalline epoxy **M1**.

## 1. Introduction

Nylon 66 has been widely used in the fields of engineering plastics and fibers because of its excellent performance due to its high strength, wear-resistance and self-lubrication. With the development of economics, new and higher requirements are proposed in every material field. Modified nylon has also gained considerable interest for its excellent mechanical strength, heat resistance, oil resistance, chemical resistance and other properties. Mason [1] and Baer [2] had obtained high impact resistance nylon. Anbarasan [3] studied chemical grafting of polyaniline onto nylon 66 fiber in different media. Yue Huang and Krumova [4,5] explored the performance and characteristics of modified nylon using microindentation technique. By adding soft phase to absorbing impact energy into the hard nylon matrix, the toughness of nylon was improved without a dramatic reduction in strength and rigidity [6–8]. DuPont and BASF, *etc*., have produced nylon 66 with high content of glass fiber, which possesses low water absorption, high rigidity, heat deflection temperature and dimensional stability [9,10]. Preparation of nanocomposite polymer using nano-composite technology is a new direction in modifying polymers. With a small amount of nano-materials such as montmorillonite nanocomposites [11,12] added to the nylon substrate, the original performances, such as strength, modulus, heat deflection temperature and even air tightness can be greatly enhanced [13]. So far, few articles about nylon modified with liquid crystalline epoxy resin were published. Liquid crystalline polymers as a new class of materials have been successfully used in the technology of polymer materials. It can form fibrils in the blends of nylon 66 and thermotropic liquid crystalline polymers and thus change the thermal behavior of the system [14]. Self-reinforcement of blends containing liquid crystalline polymer has been widely due to the special structural property. Primary and secondary amines are commonly used as curing agents to obtain epoxy resin. Nylon 66 contains plenty of primary amine which could react with epoxy monomer, thus forming an interpenetrating polymer network. Rigid rod liquid crystalline epoxy resin runs through and entangles with the nylon 66.

In this study, our work was focused on the thermal properties of nylon 66 modified with liquid crystalline epoxy resin (**M1**). 3,3′,5,5′-Tetramethylbiphenyl-4,4′-diylbis(4-(oxiran-2-ylmethoxy) benzoate) (**M1**) was synthesized with a high efficient and economical method [15]. Effects on modifying diglycidyl ether of bisphenol A (DGEBA)/4,4′-diaminodiphenyl methane (DDM) revealed that the izod notched impact strength could be enhanced by 55% by the addition of 2% **M1** compared to unmodified DGEBA blends [16,17]. With respect to its excellent performance in improving mechanic and thermal properties of ordinary commercial epoxy resin, a good performance on modifyingnylon66 (**M2**) will be a reasonable expectation.

## 2. Results and Discussion

### 2.1. Mixing Analysis

**Figure 1:**
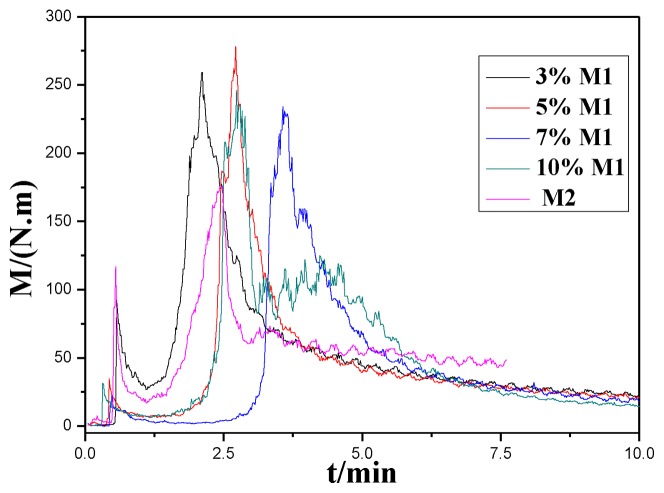
Torque *vs.* time for blends with different content of **M1** performed at 260 °C and 60 min^−1^.

**Figure 2:**
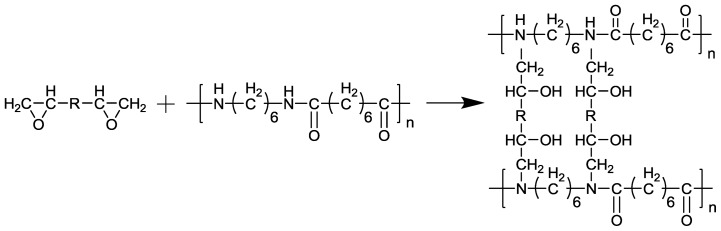
The crosslink reaction formula of nylon 66 and liquid crystalline epoxy.

A rheological test was used to evaluate thermal properties, viscosity and shear stability of thermosetting plastics consistently and accurately. Materials were mixed during feeding and torque M would change consistently along with time, which could lead us to study the rheological property and processing quality of the materials. The absolute value of torque M reflected the chemical and physical characters and apparent viscosity directly. Torque M *vs.* time reflected the degree of uniformity of the mixture. The torque M recorded during mixing is reported in Figure 1*vs.* time for the runs performed at a temperature of 260 °C and an rpm of 60 min^−1^. As time goes on, the low peaks appeared at the start of mixing, representing the completion of feeding. The onset melting points of M1 and M2 are 251.8 °C [15] and 251.2 °C respectively, so both M1 and M2 melted as soon as they were fed. The molecule structure of **M1** and **M2** are presented in Table 1 respectively. The high peaks indicate the end of cross-linking before the torque-time curve flattens. As shown in Figure 1, samples of **M2** modified with **M1** (**M1**/**M2**) show a dramatic increase of the torque, indicating that the addition of M1 led to a great increase in viscosity. The torque M decreases gradually, meaning composites have begun to melt and reache the balanced torque. Finally the temperature of the material increases to the set point after the addition of **M1**. Compared with **M2**, the torques of **M1**/**M2** blends increase at different degrees, indicating that the addition of **M1** gives rise to partial cross-linking in the system. Epoxy resin monomer contains a reactive epoxy group in the molecule chain that could react with primary and secondary amines, thus form a crosslink network structure. The epoxy in **M1** could react with amine group in the terminal of **M2**; a light crosslink network structure is formed as is presented in Figure 2. Chains of molecules begin to bind. Thus, the viscosity of the system increases, leading to the increase of torque. As the cross-linking finishes, the torque starts to decrease, reaching the balance again. The blends still stay thermoplastic.

**Figure 3:**
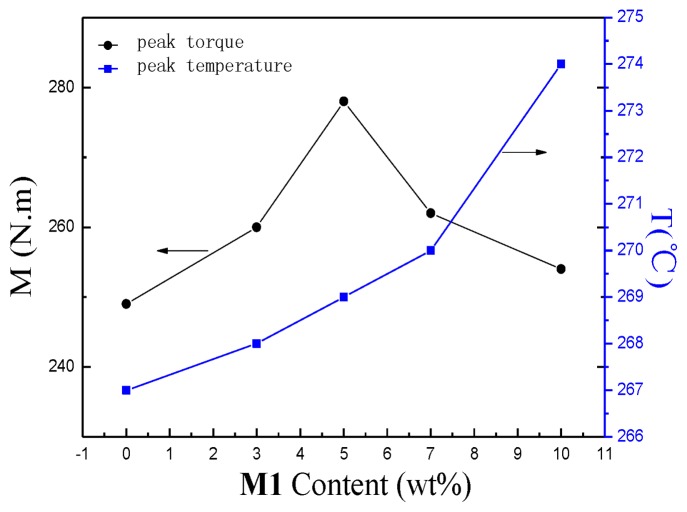
Effect of content of M1 on torque and temperature of modified system.

Peak values of torque M in Figure 1 were summarized to form the black curve line in Figure 3. The blue curve line showed the peak temperature during the melting of the mixture with different amounts of **M1**. Figure 3 describes the influence of the amount of **M1** on the torque and temperature of the modified system. As is shown, the melt temperature of modified system surpasses the setting point (260 °C) and increases gradually with the increase of the amount of **M1**. It is a proof that the reaction of **M1** and **M2** during mixing is an exothermic reaction. The peak torque increases with an increase in the amount of **M1**. When the content of **M1** reaches 5%, the peak torque reaches its extreme value and then begins to decrease. This is probably due to the reunion of **M1** when the content of **M1** surpasses 5%. So the dispersion of **M1** in **M2** becomes difficult and the opportunity to react with **M2** reduces.

### 2.2. Thermogravimetric Analysis

**Figure 4:**
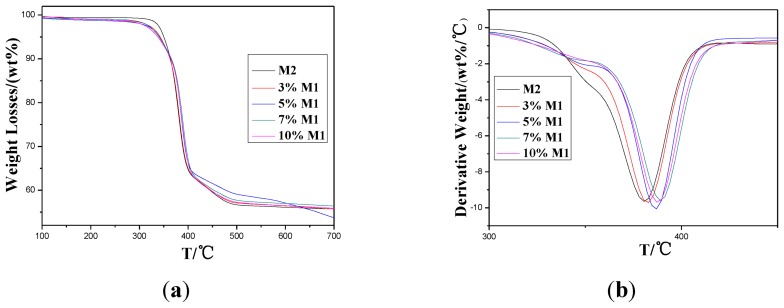
Isothermal TG curves (**a**) of blends of different content of **M1** as a function of cure temperature and DTG (**b**) curve of the compositions.

Thermal stability and thermal-degradation property were evaluated with TGA. TGA thermograms of the **M2** and **M1**/**M2** are shown in Figure 4. These test samples show a conspicuous weight loss between 350–400 °C, as seen in the figure. Thermal decomposition parameters according to Figure 4 are listed in Table 2.

As shown in Table 2, *T*_di_ and *T*_d_ were increased by 5–10 °C after small additions of **M1** to **M2** compared to **M2**, showing improvement in thermal stability. This increase may be attributed to the long range molecular orientation feature of **M1** which probably improved the thermal conductivity of the composition. Initial decomposition temperatures of different samples in Table 2 were analyzed in Figure 4.

**Figure 5:**
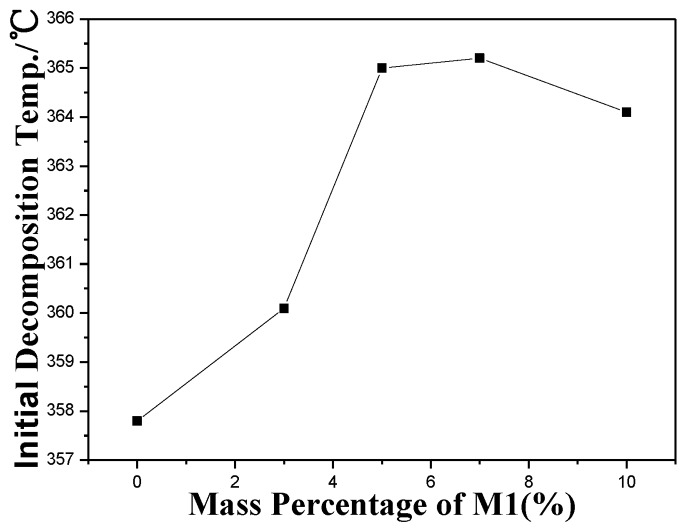
Initial decomposition temperature *vs*. compositions.

In Figure 5, another interesting phenomenon can be found. Both *T*_di_ and *T*_d_ increase with increasing **M1** content up to 7% wt, and then decrease as the **M1** content increases. The first increase of *T*_di_ and *T*_d_ may be ascribed to the formation of thermal network and the increased chance of reunion when further increase in content of **M1** after 7% wt may explain the decrease of *T*_di_ and *T*_d_.

As the main chains of aromatic polymers have an aromatic ring, they can get a high rate of carbon. The higher the degree of aromatics, the higher the rate of carbon. If the yield of carbon becomes more aromatic and tighter, its resistance to thermal decomposition at high temperature becomes stronger and more stable. According to the structure of **M1**, it has a plenty of aromatic rings which condense to aromatic carbon at high temperature; the generation of those carbons can then affect decomposition. They form an insulating layer on the surface of the polymer, making further decomposition become more difficult, so the thermal resistance of modified system improves.

### 2.3. Differential Scanning Calorimetry Analysis

**Figure 6:**
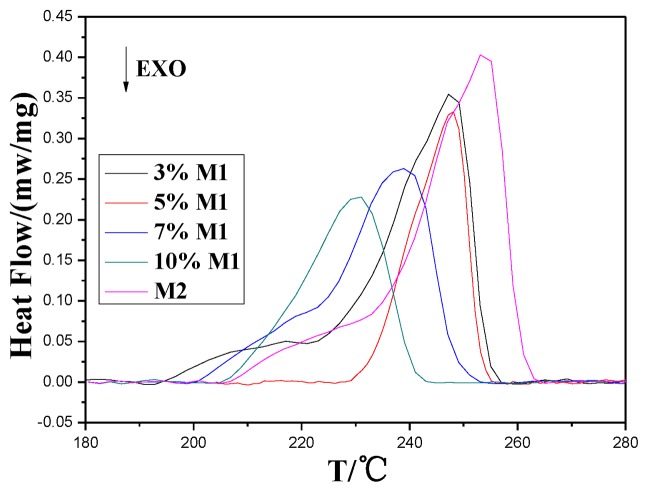
Differential Scanning Calorimetry (DSC) curves of samples at heating rate of 20 °C/min.

DSC measurement was performed to demonstrate thermal effects of the materials whose melting behavior were analyzed systematically. Figure 6 depicts the melting curves of the test samples on DSC instruments. All samples show a single peak, representing good compatibility of **M1** and **M2**. The melting point (*T*_m_), onset melting point (*T*_mo_), final melting point (*T*_mf_), melting temperature range (Δ*T*_m_, Δ*T*_m_= *T*_mf_ − *T*_mo_) and the melting enthalpy (Δ*H*_m_) can also be obtained from Figure 6. The thermodynamic parameters of each sample are reported in Table 3.

Thermodynamic parameters in Table 3 and Figure 6 show the DSC scans obtained for **M2** and **M1**/**M2** upon heating. The melting pointing(*T*_m_) and onset melting point (*T*_mo_) of **M1**/**M2** decrease with the improvement of **M1**. Compared to **M2**, the decrease of melting point of **M1**/**M2** may be put down to the hyper-dispersion of **M1** among **M2**. It damages the regularity of **M2** crystallization, resulting in the decrease of melting point. In contrast, the melting temperature range (Δ*T*_m_) [18], which indicates the degree of crystal perfection, increases. This result may be attributed to a small amount of **M1** present in the sample, which has a heterogeneous nucleation effect on PA66 (**M2**) and promotes the generation of a different degree of crystal perfection. The enthalpy of melting Δ*H*_m_ was found to decrease with the increasing content of **M1**.

### 2.4. Crystallite Morphology

**Figure 7:**
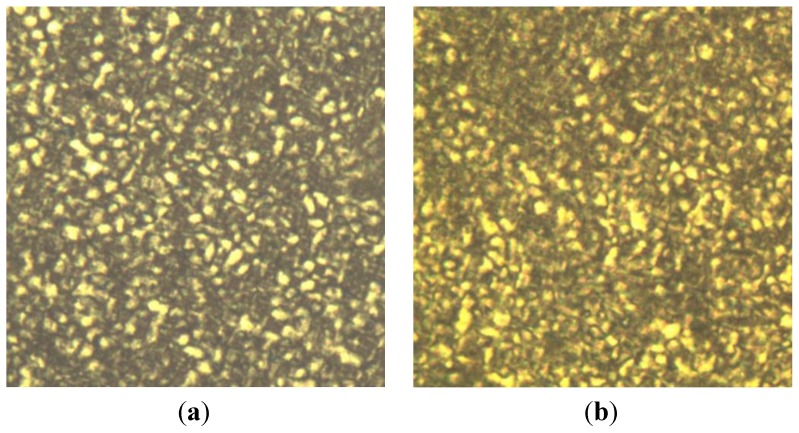
Polarized optical microscopy (POM) images of nylon 66 (**a**) and LCE-nylon 66 (**b**) after holding at 268 °C for 40 min then slowed cooled temperature. (**a**) (**b**)

POM with a hot stage was used to observe microstructure of polymer during crystallization. It is an important way to study the optical properties of crystal. Figure 7 depicts the POM images of neat **M2** and **M1** modified **M2. M2** exhibits the usual spherulitic structure characterized with Maltese cross. However, after the addition of **M1**, the sizes of spherulites clearly decrease, indicating that the structure of **M2** spherulites was partly destroyed by **M1**. Feng *et al.* studied the crystallization behavior of co-polyamide 66 (FR-PA66) containing triarylphosphine oxide (TPO) which acts as a flame retardant [19]. They also observed the size change. Other researchers also observed similar crystallization structure [20]. The polymer spherulite dimension is governed by the number of nuclei formed in a unit volume at the time of crystallization. The addition of liquid crystalline epoxy affects the crystallization rate and dimension of nylon-66 spherulite. **M1** acts as a heterogeneous nucleation agent. Meanwhile, interaction between the **M1** and **M2** molecules decreases the number of crystalline **M2** molecules and results in imperfect crystalline structures. Thus, perfect spherulites cannot form and the spherulite size decreases which indicates that **M1** groups are unfavorable for **M1** modified **M2** crystallization. These results are consistent with non-isothermal crystallization analysis.

## 3. Experimental Section

Chemical structure of **M1** employed in our study was given in Table 1. Synthesis and characterization of **M1** was reported previously by our group. Nylon 66 (**M2**) was provided by Zhejiang Xinli Plastic Co., Ltd.

**M1** and **M2** were both carefully dried at 70 °C for at least 24 h, and then blended using a HAAKE 400P Rheometer. During mixing the torque was continuously recorded. The content of **M1** in the compositions ø was 0, 3, 5, 7 and 10 wt% and the composites were prepared after mixing in the HAAKE 400P Rheometer for 15 min. Then the prepared mixtures were kept in sealed bags at room temperature before use.

Mixing of reactants was recorded by HAAKE 400P Rheometer to confirm crosslink between Functional groups. Nylon 66 particles along with **M1** was melt mixed with a screw speed of 60 r/min at a feeder temperature of 260 °C for 15 min to achieve a uniform dispersion.

Thermogravimetric analysis (TGA) was performed on a Netzsch STA 449C thermogravimetric analyzer under a nitrogen atmosphere at a heating rate of 10 °C·min^−1^ up to 800 °C. The degradation temperature of the samples was analyzed from TGA plot.

Differential Scanning Calorimetry (DSC) texting: Non-isothermal crystallization behaviors of blends of M1 and M2 were carried out using Netzsch DSC204F1 to determine if complete crystallization was obtained in samples and to observe the thermal transitions of the materials. All DSC experiments were performed at heating/cooling rate of 20 °C/min in an atmosphere of nitrogen.

Polarized optical microscopy (POM) was performed to determine the phase morphology of crystallization. Small particles of simples were spread on a round glass slide and then covered by another slide. The samples were heated to 268 °C to melt and kept at this temperature for 40 min and then cooled to ambient temperature, crystallization behaviors were observed with POM. Comparison of images of spherulite growing of pure **M2** and **M1** modified **M2** was carried out with POM.

## 4. Conclusions

The results discussed above lead to two main conclusions: First, small additions of **M1** to **M2** increase the initial decomposition temperature (*T*_di_) by 5~10 °C; second, the melting point of **M1**/**M2** blends decreases with respect to **M1**. It also decreases as the content of **M1** increases. As for the first effect, a similar effect had already been noticed by another author for blends of PA66/Amine-based multi-walled carbon nanotubes [21]. The literature data is in agreement with our present study, considering that the system studied is not the same. The evidence obtained in our study indicates that the increase of initial decomposition temperature can be interpreted as being due to the increase of thermal conductivity of the composites caused by the long range molecular orientation feature of **M1**. As for the reduction of melting point of **M1**/**M2**, the damage of regularity of **M2** caused by hyper dispersion of **M1** and the heterogeneous nucleation effect of **M1** can probably account for this discrepancy. Finally, a comparison of **M2** and **M1**/**M2** has shown that the initial decomposition temperature of **M2** increased by about 8 °C by adding 7% wt **M1** to it, whereas the melting point decreased approximately 12 °C, showing the improvement of the processing property. **M1** acted act as nucleating agents which resulted in the decrease of spherulite dimension of the crystallization, indicating the crystallization structure was partly destroyed by adding **M1**.

## Figures and Tables

**Table 1:** Structure of **M1** and **M2**.

Monomer	Constitutions Formula
**M1**	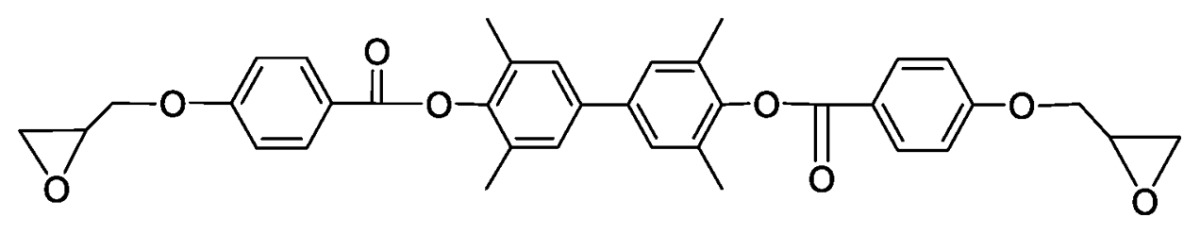
**M2**	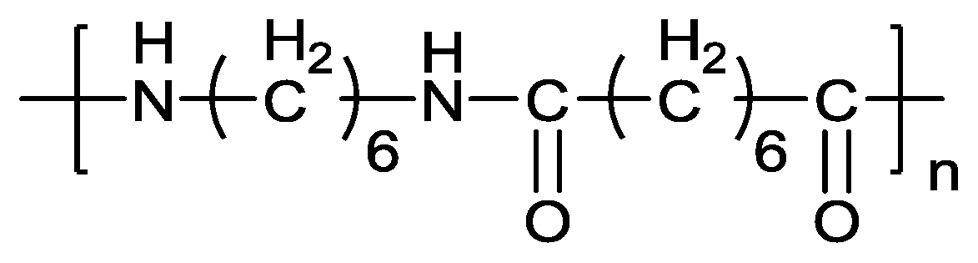

**Table 2:** Thermal decomposition parameters of composites.

ø	0	3	5	7	10
*T*_di_ (°C)	358	360	365	365	364
*T*_d_ (°C)	381	382	386	389	388
*T*_dt_ (°C)	402	402	398	407	406

ps: *T*_di_—initial decomposition temperature; *T*_d_—decomposition temperature at inflection point; *T*_dt_—terminal decomposition temperature.

**Table 3:** Thermodynamic parameters of each sample.

ø	0	3	5	7	10
*T*_m_/°C	251.2	247.2	241.5	239.1	231.1
*T*_mo_/°C	238.1	229.5	221.1	218.9	211.4
*T*_mf_/°C	260.0	254.0	248.8	247.6	240.6
*ΔT*_m_/°C	21.9	24.5	27.7	28.7	29.2
*ΔH*_m_/(J.g^−1^)	16.5	15.9	11.8	10	12.3
